# Opposing Immune-Metabolic Signature in Visceral Versus Subcutaneous Adipose Tissue in Patients with Adenocarcinoma of the Oesophagus and the Oesophagogastric Junction

**DOI:** 10.3390/metabo11110768

**Published:** 2021-11-10

**Authors:** Aisling B. Heeran, Jessica McCready, Margaret R. Dunne, Noel E. Donlon, Timothy S. Nugent, Anshul Bhardwaj, Kathleen A. J. Mitchelson, Amy M. Buckley, Narayanasamy Ravi, Helen M. Roche, John V. Reynolds, Niamh Lynam-Lennon, Jacintha O’Sullivan

**Affiliations:** 1 Trinity St. James’s Cancer Institute, Trinity Translational Medicine Institute, Department of Surgery, Trinity College Dublin, St. James’s Hospital, D08 W9RT Dublin, Ireland; heerana@tcd.ie (A.B.H.); dunnem12@tcd.ie (M.R.D.); donlonn@tcd.ie (N.E.D.); nugentti@tcd.ie (T.S.N.); Anshul.Bhardwaj@tcd.ie (A.B.); bucklea@tcd.ie (A.M.B.); nravi@stjames.ie (N.R.); reynoljv@tcd.ie (J.V.R.); lynamlen@tcd.ie (N.L.-L.); 2 Department of Biological and Physical Sciences, Assumption University, Worcester, MA 01609, USA; j.mccready@assumption.edu; 3 Nutrigenomics Research Group, School of Public Health, Physiotherapy and Sports Science, UCD Conway Institute, University College Dublin, Belfield, D04 V1W8 Dublin, Ireland; kathleen.mitchelson@ucdconnect.ie (K.A.J.M.); helen.roche@ucd.ie (H.M.R.); 4 Institute for Global Food Security, Queens University Belfast, Belfast BT9 5DL, UK

**Keywords:** adipose tissue, immune mediators, immune-metabolic signature, metabolism, oesophageal cancer

## Abstract

Oesophageal adenocarcinoma (OAC) is an exemplar model of obesity-associated cancer. Previous work in our group has demonstrated that overweight/obese OAC patients have better responses to neoadjuvant therapy, but the underlying mechanisms are unknown. Unravelling the immune–metabolic signatures of adipose tissue may provide insight for this observation. We hypothesised that different metabolic pathways predominate in visceral (VAT) and subcutaneous adipose tissue (SAT) and inflammatory secretions will differ between the fat depots. Real-time ex vivo metabolic profiles of VAT and SAT from 12 OAC patients were analysed. These samples were screened for the secretion of 54 inflammatory mediators, and data were correlated with patient body composition. Oxidative phosphorylation (OXPHOS) was significantly higher in VAT when compared to SAT. OXPHOS was significantly higher in the SAT of patients receiving neoadjuvant treatment. VEGF-A, VEGF-C, P1GF, Flt-1, bFGF, IL-15, IL-16, IL-17A, CRP, SAA, ICAM-1, VCAM-1, IL-2, IL-13, IFN-γ, and MIP-1β secretions were significantly higher from VAT than SAT. Higher levels of bFGF, Eotaxin-3, and TNF-α were secreted from the VAT of obese patients, while higher levels of IL-23 and TARC were secreted from the SAT of obese patients. The angiogenic factors, bFGF and VEGF-C, correlated with visceral fat area. Levels of OXPHOS are higher in VAT than SAT. Angiogenic, vascular injury and inflammatory cytokines are elevated in VAT versus SAT, indicating that VAT may promote inflammation, linked to regulating treatment response.

## 1. Introduction

Oesophageal cancer is the seventh most common cancer worldwide and the sixth most common cause of cancer death [1]. The two main histological subtypes of oesophageal cancer are oesophageal adenocarcinoma (OAC) and squamous cell carcinoma. OAC predominates in high-income countries, with lifestyle factors including obesity and reflux disease considered key risk factors [2]. Most patients with locally advanced OAC at presentation receive neoadjuvant chemotherapy with or without radiation prior to surgery [3]. The attainment of a complete pathological response, characterised by no viable tumour present in the resection specimen, and a proxy for improved survival outcomes is achieved in 23% of patients [4,5]. While the influence of obesity on disease risk is well-documented, the effect of obesity status on treatment response to chemotherapy and radiation therapy is poorly understood. A study by Mongan et al. suggests that overweight or obese OAC patients were more likely to have a better response to neoadjuvant chemoradiation (neo-CRT) compared to patients of normal weight [6]. This in vitro study reported that the addition of adipose conditioned media (ACM) from VAT resulted in an improved radio-response in a radioresistant OAC cell line, irrespective of the obesity status of the patient or whether or not the patient had cancer [6]. Only ACM generated from VAT from patients that received neo-CRT induced radiosensitisation in a radiosensitive OAC cell line [6]. These results suggest that radiosensitive and radioresistant tumours may have a differential response to factors secreted from VAT. It appears that, in a radioresistant OAC model, the radio-sensitising effects are due to factors secreted from VAT as opposed to cancer effects or bystander effects following cancer treatment [6]. The mechanistic basis of this enhanced response is unclear. However, it was possible to discriminate between ACM from non-cancer patients and ACM from OAC patients receiving surgery only or receiving neo-CRT, based on metabolite profiles. Higher levels of threonine, lysine, and valine and lower levels of glucose were observed in the ACM of OAC patients receiving surgery as their only treatment modality compared to their cancer-free counterparts. When compared to a cohort of OAC patients receiving neo-CRT, the ACM from the non-cancer controls had elevated levels of glucose and reduced levels of threonine, lysine, valine, isoleucine, and glycine [6]. Given the role of these metabolites in energy metabolism, it suggests that mitochondrial metabolism is altered in adipose tissue from OAC patients, and that neo-CRT can cause further alterations in this metabolic profile.

Improved response to therapy has also been reported in overweight and obese patients with metastatic melanoma, whereby obese patients treated with BRAF-targeted therapies or immune checkpoint inhibitors had improved outcomes [7]. Similarly, in a cohort of patients treated with PD-1/PD-L1 blockade, obese patients had improved outcomes, despite evidence demonstrating that obesity had negative effects on tumour progression and induced PD-1-mediated T cell dysfunction [8]. These observations were supported in a recent meta-analysis of >4000 patients, whereby a BMI > 30 kg/m^2^ was a prognostic indicator for improved response to immune checkpoint inhibitors as measured by increased overall survival and progression-free survival [9]. While the exact mechanisms underpinning these observations are largely elusive, there is evidence of increased expression of PD-1 on T cells in obese individuals, and that PD-1 levels are correlated with leptin, which is elevated in obese individuals [8].

To date, our understanding of adipose tissue metabolism and its connection to inflammation is scant. However, alterations in metabolism, angiogenesis, and inflammatory mediators have previously been shown to be implicated in altering treatment response in OAC [10]. Furthermore, it is known that alterations in factors secreted from adipose tissue from obese OAC patients result in altered cellular metabolism and mitochondrial function in OAC cells [11]. Specifically, ACM generated from obese patients had significantly higher levels of lactate and reduced levels of alanine, ethanol, isoleucine, leucine, and valine compared to ACM from a non-obese cohort of OAC patients [11]. There is a highly dynamic inter-relationship between inflammation and metabolism, wherein cellular metabolites can drive the nature of the inflammatory response, but equally, inflammatory signals can disrupt metabolism. Cellular metabolism defines immune cell functionality, wherein anaerobic glycolysis versus oxidative phosphorylation (OXPHOS) promotes a pro-inflammatory milieu [12]. Conversely, interrupting immune metabolism inhibits the capability to mount a pro-inflammatory macrophage response [13,14]. Elucidating the metabolic and inflammatory signatures of VAT and SAT may help to reveal the mechanisms underlying the observed enhanced response to neo-CRT in overweight and obese patients. This descriptive study aims to profile, for the first time, the metabolic pathways utilised in VAT and SAT of OAC patients and explore a link between real-time adipose tissue energy metabolism profiles and inflammatory signatures. 

We hypothesise that different metabolic pathways will be present and functional in VAT compared to SAT, and that the secretome of VAT will contain higher levels of inflammatory mediators compared to the SAT secretome. This study profiles the immune-metabolic signature of VAT and SAT in OAC patients for the first time. Gaining an understanding of the metabolic profile of VAT and SAT and its connections with the inflammatory secretome may begin to unravel the molecular mechanisms underlying the improved treatment response observed in overweight and obese OAC patients. 

## 2. Results

### 2.1. Metabolic Profiles Differ in VAT and SAT

**Figure 1:**
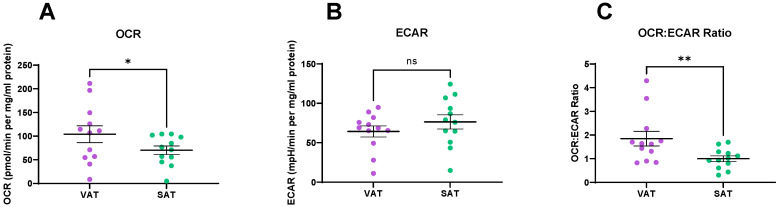
Visceral adipose tissue (VAT) and subcutaneous adipose tissue (SAT) have different metabolic profiles. (**A**) VAT has higher rates of oxidative phosphorylation (OXPHOS) compared to SAT. (**B**) There is a trend towards higher levels of glycolysis in SAT compared to VAT. (**C**) The OCR:ECAR ratio is significantly higher in VAT compared to SAT. Statistical analysis was performed using a Wilcoxon signed rank test. All data expressed as mean ± SEM. *n* = 12, ** *p* < 0.01, * *p* < 0.05, ns = non-significant.

To determine if metabolic profiles differed in VAT and SAT, we measured oxygen consumption rate (OCR) and extracellular acidification rate (ECAR), measures of OXPHOS and glycolysis, respectively, using a Seahorse XFe24 analyser. Interestingly, OCR, a measure of OXPHOS was significantly higher in VAT compared to matched SAT (*p* = 0.01) (Figure 1A). ECAR, a measure of glycolysis, demonstrated a trend towards higher levels in SAT when compared to VAT, although this did not reach statistical significance (*p* = 0.09) (Figure 1B). OCR:ECAR ratio was significantly higher in VAT when compared to SAT, indicating a greater dependence on OXPHOS in the VAT (*p* = 0.003) (Figure 1C). 

**Figure 2:**
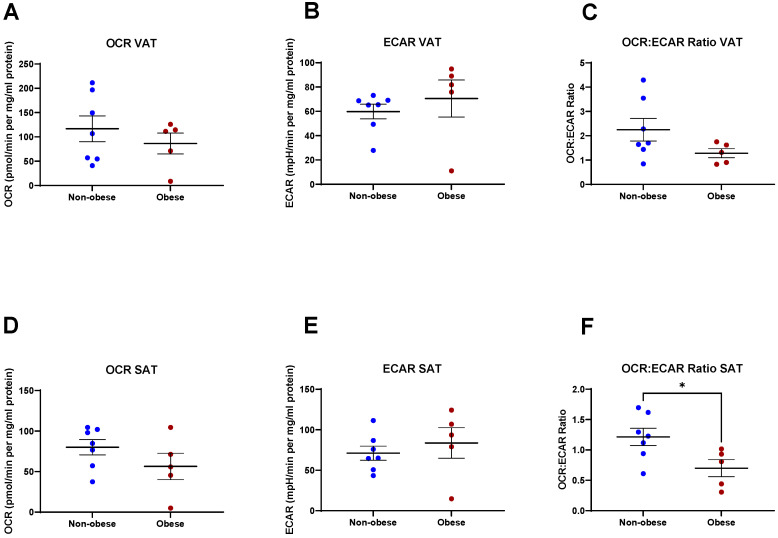
Metabolic analysis of visceral adipose tissue (VAT) and subcutaneous adipose tissue (SAT) by obesity status. There was no significant difference in (**A**) oxygen consumption rate (OCR), (**B**) extracellular acidification rate (ECAR), and (**C**) OCR:ECAR ratio in VAT between non-obese and obese oesophageal adenocarcinoma (OAC) patients. There was no significant difference in (**D**) OCR or (**E**) ECAR in SAT between non-obese and obese OAC patients. (**F**) OCR:ECAR ratio was significantly reduced in SAT of obese compared to non-obese OAC patients. Statistical analysis was performed using a Mann–Whitney U-test. All data expressed as mean ± SEM. *n* = 7 for non-obese, *n* = 5 for obese. * *p* < 0.05.

Given the tight association between OAC and obesity, we compared the metabolic profile of VAT and SAT in obese patients with those of non-obese patients and did not find any significant differences between levels of OCR, ECAR, or OCR:ECAR ratio in the VAT, using visceral fat area (VFA) as a measure of obesity (*p* > 0.05). We found a significantly reduced OCR:ECAR ratio in SAT of patients that were obese (*p* = 0.03) (Figure 2). 

**Figure 3:**
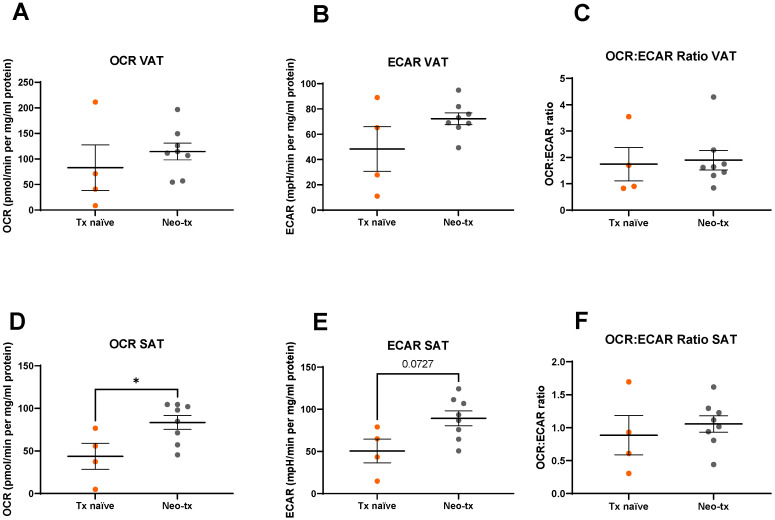
Metabolic analysis of visceral adipose tissue (VAT) and subcutaneous adipose tissue (SAT) by treatment modality. There was no significant difference in (**A**) oxygen consumption rate (OCR), (**B**) extracellular acidification rate (ECAR), or (**C**) OCR:ECAR ratio in VAT between the treatment-naïve cohort and those receiving cytotoxic neoadjuvant therapy. (**D**) OCR was significantly higher in SAT in patients receiving cytotoxic neoadjuvant therapy compared to the treatment-naïve cohort. (**E**) There was no significant difference in ECAR in SAT between patients receiving cytotoxic neoadjuvant therapy compared to the treatment-naïve cohort. (**F**) OCR:ECAR ratio was not significantly different in SAT between the treatment-naïve cohort and those receiving cytotoxic therapy. Statistical analysis was performed using a Mann–Whitney U-test. All data expressed as mean ± SEM. *n* = 4 for treatment-naïve (tx-naïve) cohort, *n* = 8 for cytotoxic neoadjuvant therapy (neo-tx) cohort. * *p* < 0.05.

We also examined the effect of cytotoxic treatment on metabolic profiles in VAT and SAT in our cohort of OAC patients and found that there were no significant differences in metabolic profiles in VAT based on whether patients received cytotoxic therapy or not (*p* > 0.05) (Figure 3A–C). Patients receiving neoadjuvant chemotherapy with or without radiation therapy had significantly higher levels of OCR (*p* = 0.04) and a trend towards higher ECAR (*p* = 0.07) in their SAT compared to the cohort of patients that received surgery only. OCR:ECAR ratio was not significantly different between the treatment-naïve cohort and the cohort that received neoadjuvant treatment (*p* > 0.05) (Figure 3D–F). 

### 2.2. Comparison of the Protein Secretome between VAT and SAT

To assess if inflammatory, cytokine, chemokine, vascular injury, and angiogenic secretions differed between VAT and SAT, MSD multiplex ELISAs were performed assessing the secretion of 54 mediators using ACM from VAT and SAT. A readout was obtained for 51 out of the 54 mediators, with the remainder being outside the limit of detection of the assay. Of the 51 detected mediators, 16 were secreted at significantly higher levels from VAT, when compared to SAT, namely FGF(basic), Flt-1, P1GF, VEGF-A, VEGF-C, CRP, SAA, ICAM-1, VCAM-1, IL-15, IL-16, IL-17A, IFN-γ, IL-13, IL-2, and MIP-1β. VEGF-D showed a trend towards higher levels being secreted from SAT compared to VAT, though this did not reach statistical significance. 

### 2.3. Angiogenic Factors Are Secreted at Significantly Higher Levels from VAT, When Compared to SAT

**Figure 4:**
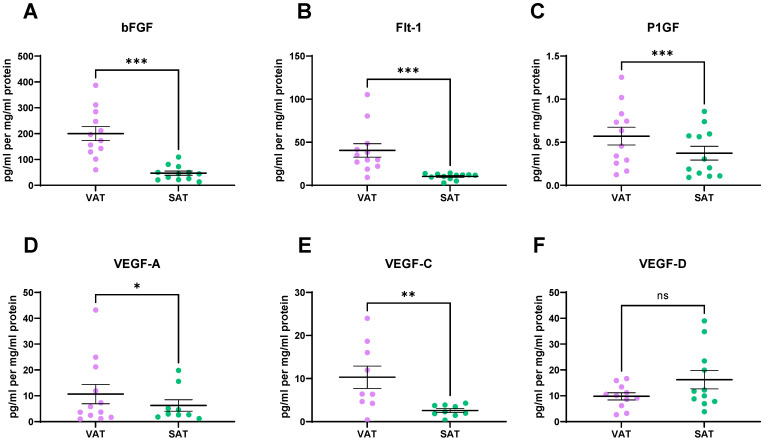
Angiogenic factors are secreted at higher levels from visceral adipose tissue (VAT), when compared to subcutaneous adipose tissue (SAT). Significantly higher levels of (**A**) bFGF, (**B**) Flt-1, (**C**) P1GF, (**D**) VEGF-A and (**E**) VEGF-C are secreted from VAT, when compared to SAT. (**F**) VEGF-D is showing a trend towards elevated levels in SAT compared to VAT. Statistical analysis was performed using a Wilcoxon signed rank test. All data expressed as mean ± SEM. *n* = 12 for bFGF, Flt-1 and P1GF, *n* = 11 paired samples for VEGF-D, *n* = 9 for VEGF-A and VEGF-C. *** *p* < 0.001, ** *p* < 0.01, * *p* < 0.05, ns = non-significant.

Among the 16 factors secreted at higher levels from VAT, 5 of these factors are angiogenic markers; bFGF (*p* = 0.0005), Flt-1 (*p* = 0.001), P1GF (*p* = 0.0005), VEGF-A (*p* = 0.01), and VEGF-C (*p* = 0.003) (Figure 4A–E). VEGF-D is also an angiogenic marker and is showing a trend towards secretion at higher levels from SAT compared to VAT (*p* = 0.053) (Figure 4F). 

### 2.4. Vascular Injury Markers Were Found at Significantly Higher Levels in the VAT Secretome Compared to the SAT Secretome

**Figure 5:**
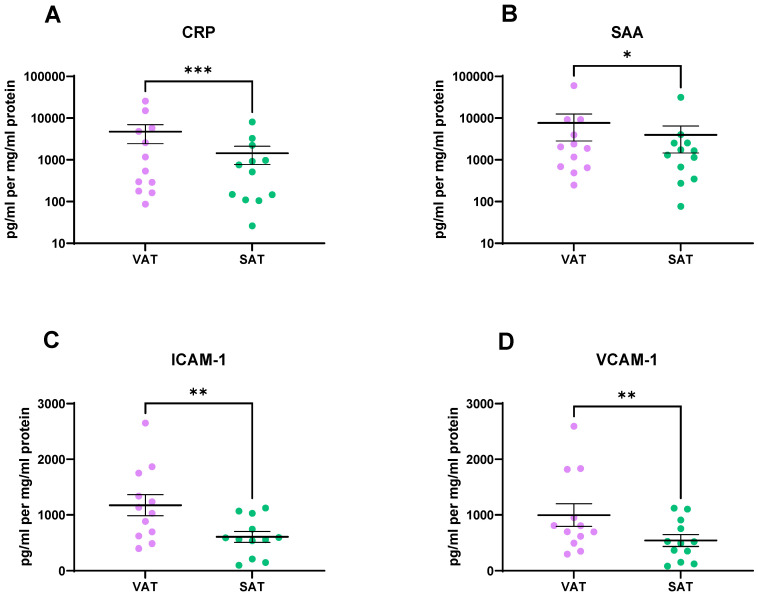
Vascular injury secretions are higher in the visceral adipose tissue (VAT) secretome when compared to the subcutaneous adipose tissue (SAT) secretome. (**A**) CRP, (**B**) SAA, (**C**) ICAM-1, and (**D**) VCAM-1 are secreted at significantly higher levels from VAT compared to SAT. Statistical analysis was performed using a Wilcoxon signed rank test. All data expressed as mean ± SEM. *n* = 12, *** *p* < 0.001, ** *p* < 0.01, * *p* < 0.05.

An MSD vascular injury assay was used to assess if there was a difference in vascular injury markers between the VAT and SAT secretomes. Vascular injury secretions were found at significantly higher levels in the secretome of the VAT compared to SAT. Specifically, CRP (*p* = 0.0005), SAA (*p* = 0.01), ICAM-1 (*p* = 0.002), and VCAM-1 (*p* = 0.001) (Figure 5A–D) were secreted at significantly higher levels from the VAT compared to SAT.

### 2.5. VAT Secretes Higher Levels of Inflammatory Mediators, Cytokines, and Chemokines Compared to SAT

**Figure 6:**
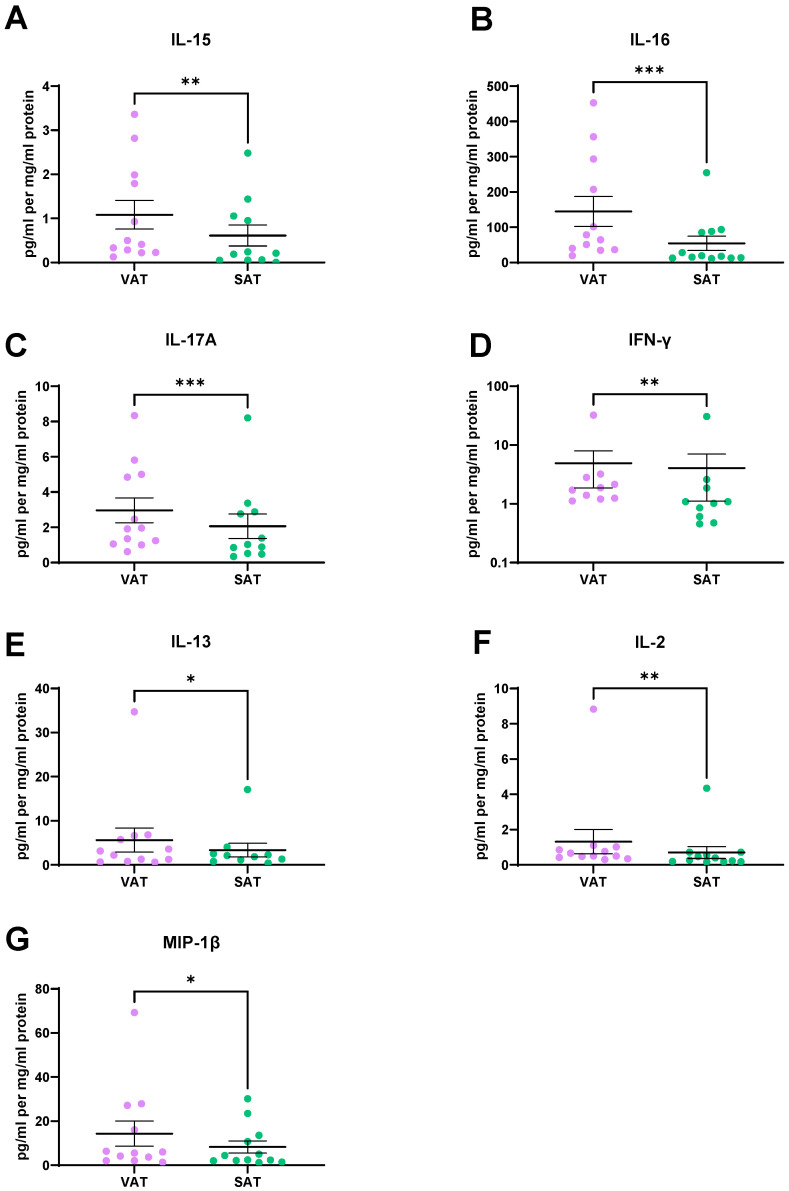
Inflammatory, cytokine, and chemokine secretions are higher in the visceral adipose tissue (VAT) secretome when compared to subcutaneous adipose tissue (SAT) secretome. There were significantly higher levels of (**A**) IL-15, (**B**) IL-16, (**C**) IL-17A, (**D**) IFN-γ, (**E**) IL-13, (**F**) IL-2, and (**G**) MIP-1β secreted from VAT when compared to SAT. All data expressed as mean ± SEM. Statistical analysis was performed using a Wilcoxon signed rank test. *n* = 12 paired samples for IL-16, IL-2 and MIP-1β, *n* = 11 paired samples for IL-17A and IL-15, *n* = 10 paired samples for IFN-γ and IL-13. *** *p* < 0.001, ** *p* < 0.01, * *p* < 0.05.

To test the hypothesis that cytokine secretion would differ between VAT and SAT, we screened the ACM for the secretion of 18 cytokines and 10 chemokines using the MSD multiplex system. All chemokines and 15 of the 18 cytokines were within the detection range of the assay. IL-15 (*p* = 0.002), IL-16 (*p* = 0.0005), and IL-17A (*p* = 0.001) (Figure 6A–C) were secreted at significantly higher levels from VAT compared to SAT. Significantly higher levels of IFN-γ (*p* = 0.003), IL-13 (*p* = 0.02), and IL-2 (*p* = 0.009) (Figure 6D–F) were found in the VAT secretome compared to the SAT secretome. MIP-1β was secreted at significantly higher levels from VAT compared to SAT (*p* = 0.03) (Figure 6G).

### 2.6. Secreted Factors from VAT and SAT Are Altered in Obese Patients

**Figure 7:**
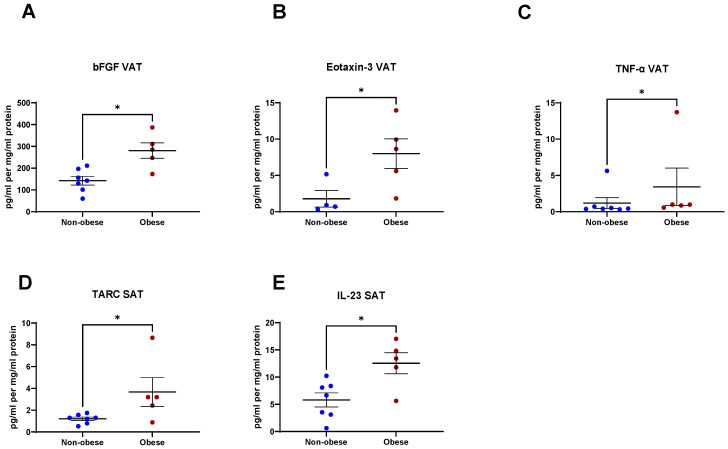
Secreted factors are altered in the visceral adipose tissue (VAT) and subcutaneous adipose tissue (SAT) based on the obesity status of the patient. (**A**) bFGF, (**B**) Eotaxin-3, and (**C**) TNF-α are secreted at significantly higher levels from the VAT of obese patients compared to non-obese patients. (**D**) TARC and (**E**) IL-23 are secreted at significantly higher levels from the SAT of obese patients compared to non-obese patients. Statistical analysis was performed using a Mann–Whitney U-test. All data expressed as mean ± SEM. *n* = 5 for obese patients, *n* = 7 for non-obese patients, *n* = 4 for non-obese patients for Eotaxin-3. * *p* < 0.05.

Having observed significantly higher levels of inflammatory secretions from VAT when compared to SAT, we examined whether obesity alters the secretion of these factors from VAT. Significantly higher levels of bFGF (*p* = 0.01), Eotaxin-3 (*p* = 0.03), and TNF-α (*p* = 0.04) were found in the secretome of VAT from obese patients compared to non-obese patients (Figure 7A–C). Similarly, we investigated if obesity is associated with altered secretion of inflammatory mediators from SAT. Elevated levels of TARC (*p* = 0.04) and IL-23 (*p* = 0.03) were found in the secretome of SAT from patients that were obese compared to their non-obese counterparts (Figure 7D,E). There were no significant differences in the levels of any other secreted inflammatory mediator from either VAT or SAT between obese and non-obese patients (data not shown).

### 2.7. Linking Energy Metabolism to Secreted Factors in VAT and SAT to Derive an Immune-Metabolic Signature

To examine the relationship between adipose tissue metabolism and secreted inflammatory mediators, we investigated the correlation between metabolic parameters and secreted factors. Table 1 outlines the relationship between the factors. TNF-β correlated with OCR in VAT (R = 0.7667, *p* = 0.02). ECAR correlated with CRP (R = 0.7203, *p* = 0.01) and P1GF (R = 0.6294, *p* = 0.03), while Eotaxin (R = −0.8667, *p* = 0.004) was found to be inversely correlated with OCR:ECAR ratio and TNF-β (R = 0.7167, *p* = 0.03) positively correlated with OCR:ECAR ratio. 

The link between energy metabolism and secreted inflammatory mediators differed between VAT and SAT. IL−2 (R = −0.5874, *p* = 0.04) and Eotaxin-3 (R = −0.7857, *p* = 0.04) inversely correlated with OCR in SAT. There was an inverse correlation between ECAR and IFN-γ (R = −0.7817, *p* = 0.01), IL-17D (R = −0.6294, *p* = 0.03), and IL-8 (R = −1, *p* = 0.01) and a positive correlation between ECAR and ICAM-1 (R = 0.6154, *p* = 0.03), bFGF (R = 0.6294, *p* = 0.03), and VEGF-D (R = 0.7455, *p* = 0.01). OCR:ECAR ratio in SAT correlated inversely with the secretion of IP-10 (R = −0.5874, *p* = 0.04) and Tie-2 (R = −0.8857, *p* = 0.03) from SAT (Table 1).

### 2.8. Linking Anthropometric Parameters to Secreted Factors in VAT and SAT 

We identified correlations between secretions of different analytes from VAT and anthropometric parameters. Subcutaneous fat area (SFA) was positively correlated with Eotaxin (R = 0.8333, *p* = 0.008). Skeletal muscle was inversely correlated with Eotaxin (R = −0.7167, *p* = 0.03), MCP-1 (R = −0.5874, *p* = 0.04), IL-12p70 (R = −0.6014, *p* = 0.04), IL-1β (R = −0.8571, *p* = 0.02), IL-6 (R = −0.6084, *p* = 0.03), and IL-8 (R = −0.5874, *p* = 0.04). Intermuscular fat (IMF) correlated with bFGF (R = 0.6182, *p* = 0.04), Eotaxin-3 (R = 0.7857, *p* = 0.02), and IL-8 (R = 0.8810, *p* = 0.007). Visceral fat area (VFA) correlated with bFGF (R = 0.6503, *p* = 0.02) and VEGF-C (R = 0.75, *p* = 0.02) (Table 2). Skeletal muscle significantly inversely correlated with VEGF-A secretion from SAT (R = −0.7667, *p* = 0.02), and IMF correlated with TNF-α secretion from SAT (R = 0.6727, *p* = 0.02) (Table 3).

## 3. Discussion

This study demonstrates, for the first time, that Seahorse technology provides a useful tool for the analysis of real-time metabolic profiles in fresh human adipose tissue from OAC patients. We also describe, for the first time, the real-time metabolic profiles of both VAT and SAT from OAC patients. We have shown that levels of OXPHOS are significantly higher in the VAT compared to SAT of OAC patients. In addition, angiogenic markers, inflammatory markers, and cytokine secretions are significantly enhanced from VAT compared with SAT. We found positive correlations between factors secreted from VAT and anthropometric measurements, including a correlation between angiogenic factors such as bFGF, VEGF-C, and VFA. OAC is an obesity-related cancer with visceral adiposity and associated metabolic syndrome, particularly incriminated in carcinogenesis, but whether the cancer biology of established tumours is altered by adipose tissue has not heretofore been studied. Intriguingly, obesity may enhance responses to chemotherapy and radiation therapy for OAC [6], and this analysis of a differential metabolic and immunoinflammatory profile between VAT and SAT may provide novel insights into key underlying mechanisms. 

To date, studies investigating the metabolic profile of adipose tissue have largely been limited to obese patients undergoing bariatric surgery. We have elucidated the energy metabolism profiles of VAT and SAT in OAC patients for the first time in fresh fat tissue and have shown that OXPHOS is higher in VAT compared to SAT. These results are in line with a study comparing the metabolic profiles of VAT and SAT in adipose tissue from obese patients undergoing bariatric surgery, whereby levels of OXPHOS were higher in VAT compared to SAT when normalised per weight of tissue [15]. Interestingly, however, VAT had twice the number of mitochondria per milligram of tissue compared to SAT, but VAT also had double the amount of cells per milligram of tissue as SAT [15]. Since this study population fell into the category of morbidly obese with a mean BMI of 40.7 kg/m^2^, it is unknown whether these results can be extrapolated to a population with a lower BMI. Even though OAC is an obesity-associated disease, the mean BMI of the patient cohort in our study is 25.5 kg/m^2^ and therefore is considerably lower than in the aforementioned study. Despite this, the same trend was observed. Moreover, analysis of the metabolic profiles of VAT did not differ based on the obesity status of the patient, using VFA as a determinant of obesity. The similarity in findings suggest that these pathways are not dependent on the tumour being present and may apply to other obesity-associated cancers and obesity-related diseases. 

Profiling the secretome of VAT and SAT in our cohort of OAC patients demonstrated that a panel of angiogenic markers including VEGF-A, bFGF, P1GF, VEGF-C, and Flt-1 were secreted at higher levels from the VAT compared to the SAT. These results are in line with a previous study by Lysaght et al. [16], which found that VEGF was significantly higher at both the protein levels in the secretome and the RNA level from VAT compared to SAT. Flt-1 (VEGFR1) is thought to play a role in obesity-related tumour progression that is unrelated to the angiogenic process. Flt-1^TK-/-^ mice exhibit an altered immune phenotype and a shift in tumour associated macrophages from an M2 to M1 phenotype [17]. Therefore, it may be possible that certain angiogenic factors influence the adipose microenvironment and the phenotype of infiltrating immune cells which may, in turn, impact tumour progression and treatment response. Of note, P1GF is a ligand for Flt-1. Deletion of P1GF in a mouse model reproduced the effects observed upon Flt-1 deletion with altered phenotype of infiltrating immune cells [17]. Guiu et al. reported VFA as being an independent predictor of response to first-line anti-angiogenic treatment with bevacizumab [18]. Bevacizumab is a monoclonal antibody that binds to VEGF and blocks VEGF interacting with the VEGF receptors. It inhibits tumour growth by normalising tumour vasculature and prevents new vasculature forming [19]. However, it is not clear if the observed association between VFA and bevacizumab response is due to enhanced production of angiogenic factors in VAT, increased volume of distribution of the drug, or a combination of both [18]. The inter-connectivity between adipose tissue and angiogenesis has previously been reported in mouse models, whereby adipose tissue mass has been shown to be regulated by vasculature with anti-angiogenic treatment inducing weight loss [20]. Furthermore, our data indicates a positive correlation between the secretion of angiogenic factors from VAT and VFA. bFGF and VEGF-C secretion from VAT correlated with VFA. Blood vessel density has previously been reported as being higher in VAT than SAT when normalised to the area of fat examined [21]; this may support the elevated levels of angiogenic factors we observed in VAT compared to in SAT. The angiogenic potential of VAT compared to SAT is unclear, with conflicting results reported in the literature. Murine models have demonstrated higher VEGF in VAT than in SAT [22], whereas the opposite has been reported in humans [23]. 

Vascular injury markers CRP, SAA, ICAM-1, and VCAM-1 were found to be significantly higher in VAT than in SAT. Exposure of HUVECs to ACM resulted in increased adhesion of monocytes to the endothelium while simultaneously increasing expression of VCAM-1 and ICAM-1 in endothelial cells, two important factors in the adhesion process [24]. Numerous studies have reported a positive association between VAT levels and serum CRP levels in populations of various ethnicities [25,26]. Circulating SAA levels were assessed in a cohort of bariatric surgery patients both pre- and post-surgery. SAA levels decreased post-operatively with a concomitant decrease in circulating hsCRP and reduction in BMI [27]. SAA mRNA levels were found to be significantly higher in SAT compared to VAT [27].

A further difference between VAT and SAT is the augmented inflammatory cytokine profile for IL-2, IL-13, and IFN-γ in VAT. The immune phenotype of adipose tissue, attributable to the stroma vascular fraction or non-fat cell components of adipose tissue, is well documented and is responsible for the secretion of certain adipokines and inflammatory cytokines [14,28]. The enhanced secretion of pro-inflammatory markers from VAT compared to SAT is unsurprising given the finding that there are twice the number of infiltrating macrophages in VAT compared to SAT; therefore, it may be possible that the source of the pro-inflammatory secretions may be the infiltrating macrophages [29]. IFN-γ transcripts were previously reported to be 8.2-fold higher in VAT than in SAT [30]. The same study reported an increased frequency of IFN-γ-producing NK cells in VAT relative to in SAT [30]. Therefore, it may be possible that the non-fat cell component is the source of these pro-inflammatory cytokines as has been reported previously. IL-13 is a cytokine involved in insulin resistance [31,32] and elevated serum levels of IL-13 have been associated with insulin resistance [33]. Furthermore, IL-13 levels have been found to correlate strongly with BMI and waist circumference, with a weaker correlation between IL-13 and waist-to-hip ratio and body-fat percentage [33]. While it was previously thought that adipokines were secreted from the fat cell component of adipose tissue, it is now known that the non-fat cell component is responsible for the secretion of a plethora of adipokines, including ICAM-1, VCAM-1, CRP, and VEGF [28]. Greater numbers of macrophages accumulate in VAT compared to SAT [29], and this may account, at least partially, for the observed elevated levels of inflammatory mediators. 

IL-15, IL-16, and IL-17A are significantly higher in the secretome of VAT than in that of SAT. IL-15 knock-out has been previously shown to protect against obesity and diet-induced insulin resistance. Furthermore, the expression of inflammatory cytokines including TNF-α and IL-6 was reduced in the white adipose tissue of IL-15 knock-out mice [34]. IL-16 has been shown to be secreted at higher levels from mast cells of obese patients compared to lean patients [35]. IL-17A has been implicated in obesity in recent years, with obese women exhibiting elevated levels of IL-17 compared to their non-obese counterparts [36]; therefore, it is unsurprising that levels are higher in the secretome of VAT compared to SAT. 

We analysed the secretome of VAT and SAT based on the obesity status of the patient and found significantly higher levels of bFGF, Eotaxin-3, and TNF-α secreted from the VAT of obese patients than from that of non-obese patients. Moreover, higher levels of TARC and IL-23 were secreted from the SAT of obese patients compared to from that of the non-obese cohort. These results suggest that obesity status may alter the levels of inflammatory mediators produced by adipose tissue. Given that OAC is an obesity-associated cancer, these findings may offer insight into the alterations in levels of systemic inflammatory factors in obese patients. 

Our data indicate that patients lost a significant amount of skeletal muscle and overall fat-free mass from time of diagnosis to time of surgery (Supplementary Table S1). This phenomenon has been reported previously in oesophageal cancer patients by Guinan et al. [37] and Elliott et al. [38]. Sarcopenia, which is the loss of skeletal muscle mass, is associated with adverse outcomes such as dose-limiting toxicity in neoadjuvant treatment [38,39], disease progression, and post-operative complications [40]. Cancer cachexia is a progressive wasting condition characterised by sarcopenia with or without loss of fat mass and is characterised by systemic inflammation [41]. Therefore, we sought to identify if factors secreted from VAT or SAT correlated with measurements of body composition. There was an inverse correlation between skeletal muscle and the inflammatory markers IL-1β, IL-6, and IL-8. IL-6 has been implicated in the systemic inflammatory response of cachexia [42]. It is not overly surprising therefore that there was an inverse correlation between IL-6 secretion from VAT and skeletal muscle in this cohort. IL-8 serum concentrations have been shown to be elevated in cachectic pancreatic cancer patients [43]. Interestingly, elevated IL-1β in the SAT has been reported in cachectic gastrointestinal cancer patients [44].

To the best of our knowledge, this is the first study to profile an immune-metabolic signature of both VAT and SAT in a cohort of oesophageal cancer patients. Both compartments are markedly different and offer insights as to how adipose tissue and the obese microenvironment may both promote carcinogenesis and impact tumour biology and clinical responses to standard therapy. Further study of VAT in particular and its impact and clinical relevance to standard therapies, as well as anti-angiogenic and immunotherapy, may reveal novel insights that may be therapeutically applied in the clinic. 

## 4. Materials and Methods

### 4.1. Patient Recruitment

Histologically confirmed OAC and oesophagogastric junction (OGJ) carcinoma patients were prospectively recruited to this study between October 2018 and June 2019. All patient data is summarised in Supplementary Table S2. Informed written patient consent was obtained for the use of patient tissue in this study. Ethical approval was granted by the St. James’s Hospital/AMNCH Research Ethics Committee. Patient data was pseudo-anonymised prior to sample access. All samples were coded with a unique biobank number by the biobank manager. VAT and SAT were excised at the beginning of the surgical resections. Adipose tissue resections were obtained from the midline excision. Omentum, representing VAT, was excised from around the greater curvature of the stomach. VFA was calculated by a radiologist using diagnostic computed tomography scans and patients with a VFA greater than 163.8 cm^2^ (males) and 80.1 cm^2^ (females) were classified as obese [45]. Representative images of scans from a viscerally obese sarcopenic patient and a non-viscerally obese non-sarcopenic patient are shown in Supplementary Figure S1.

### 4.2. OCR and ECAR Measurements in Visceral and Subcutaneous Adipose Tissue

VAT and SAT were cut into small pieces (~20–25 mg) and plated one piece per well in 1 mL of M199 (supplemented with 0.1% gentamicin) media in a 24-well islet cell microcapture plate (Agilent Technologies, Santa Clara, CA, USA). The adipose tissue was allowed to equilibrate for 20 min at 37 °C and 5% CO_2_/95% air in the islet cell microcapture plate prior to insertion of an islet screen above the adipose tissue to prevent the tissue coming in contact with the sensors during the assay. Prior to the assay, the Seahorse XFe24 cartridge plate was hydrated for 1 h at 37 °C in a CO_2_-free incubator. Three measurements of OCR and ECAR, measures of OXPHOS and glycolysis, respectively, were taken over 24 min consisting of three repeats of mix (3 min)/wait (2 min)/measure (3 min) to establish basal OXPHOS and glycolysis levels. Upon completion of the assay, the M199 media was harvested and stored at −80 °C, and the adipose tissue was harvested and snap frozen in liquid nitrogen prior to storage at −80 °C. All measurements were normalised to protein content of the adipose tissue. OCR and ECAR measurements were calculated as the average readout of each piece of VAT and SAT over the three readings. 

### 4.3. Protein Isolation and Quantification from Adipose Tissue

Adipose tissue samples were placed on ice, and 200 μL of RIPA buffer supplemented with 1 PhosSTOP and 1 Complete Mini protease inhibitor cocktail tablet per 10 mL were added to each sample. Samples were ruptured using a metal bead and the tissue lyser at 25 Hz for 2 min. Samples were transferred to an Eppendorf tube and centrifuged at 13,000× *g* for 20 min at 4 °C. Isolated protein was quantified using BCA assay (Pierce) as per manufacturer’s instructions. 

### 4.4. Mesoscale Discovery (MSD) 54-Plex ELISA

To assess angiogenic, vascular injury, inflammatory, cytokine, and chemokine secretions in ACM from VAT and SAT, a 54-plex ELISA kit spread across 7 plates was used (Meso Scale Diagnostics, Maryland, MD, USA). The 54-multiplex kit was used to quantify the secretions of CRP, Eotaxin, Eotaxin-3, FGF(basic), Flt-1, GM-CSF, ICAM-1, IFN-γ, IL-10, IL-12/IL-23p40, IL-12p70, IL-13, IL-15, IL-16, IL-17A, IL-17A/F, IL-17B, IL-17C, IL-17D, IL-1RA, IL-1α, IL-1β, IL-2, IL-21, IL-22, IL-23, IL-27, IL-3, IL-31, IL-4, IL-5, IL-6, IL-7, IL-8, IL-8 (HA), IL-9, IP-10, MCP-1, MCP-4, MDC, MIP-1α, MIP-1β, MIP-3α, PlGF, SAA, TARC, Tie-2, TNF-α, TNF-β, TSLP, VCAM-1, VEGF-A, VEGF-C, and VEGF-D from VAT and SAT using the ACM harvested from VAT and SAT following the Seahorse assay. Secretion data for all factors was normalised appropriately to adipose tissue protein content using the BCA assay (Pierce). All assays were run as per manufacturer’s recommendations, with an alternative protocol overnight supernatant incubation being used for all assays except vascular injury and angiogenesis. MSD analysis was performed on ACM from 5 randomly selected pieces of VAT and SAT, and the average of the five pieces was used as the readout for that patient sample.

### 4.5. Statistical Analysis

GraphPad Prism 9 software was used to perform statistical analysis. All data are expressed as mean ± SEM. Data were analysed by Wilcoxon signed rank test or Mann–Whitney U-test, as stated in each figure legend. Correlation analysis was performed using Spearman’s correlation coefficient. Statistical significance was considered at *p* < 0.05.

## Figures and Tables

**Table 1:** Correlation of metabolic parameters with secreted factors from VAT and SAT.

Adipose Depot	Metabolic Parameter	Factor	R Value	*p* Value	*n*
VAT	OCR	TNF-β	0.7667	0.02	9
VAT	ECAR	CRP	0.7203	0.01	12
VAT	ECAR	P1GF	0.6294	0.03	12
VAT	OCR:ECAR	TNF-β	0.7167	0.03	9
VAT	OCR:ECAR	Eotaxin	−0.8667	0.004	9
SAT	OCR	IL-2	−0.5874	0.04	12
SAT	OCR	Eotaxin-3	−0.7857	0.04	7
SAT	ECAR	IFN-γ	−0.7818	0.01	10
SAT	ECAR	IL-17D	−0.6294	0.03	12
SAT	ECAR	ICAM-1	0.6154	0.03	12
SAT	ECAR	IL-8	−1	0.01	5
SAT	ECAR	bFGF	0.6294	0.03	12
SAT	ECAR	VEGF-D	0.7455	0.01	11
SAT	OCR:ECAR	IP-10	−0.5874	0.04	12
SAT	OCR:ECAR	Tie-2	−0.8857	0.03	6

**Table 2:** Anthropometric parameters correlating with secreted factors from VAT.

Anthropometric Parameter	Factor	R Value	*p* Value	*n*
SFA	Eotaxin	0.8333	0.008	9
Skeletal muscle	Eotaxin	−0.7167	0.03	9
Skeletal muscle	MCP-1	−0.5874	0.04	12
Skeletal muscle	IL12p70	−0.6014	0.04	12
Skeletal muscle	IL-1β	−0.8571	0.02	7
Skeletal muscle	IL-6	−0.6084	0.03	12
Skeletal muscle	IL-8	−0.5874	0.04	12
IMF	bFGF	0.6182	0.04	11
IMF	Eotaxin-3	0.7857	0.02	8
IMF	IL-8	0.8810	0.007	8
VFA	bFGF	0.6503	0.02	12
VFA	VEGF-C	0.75	0.02	9

Abbreviations: SFA—subcutaneous fat area, IMF—intermuscular fat, VFA—visceral fat area.

**Table 3:** Anthropometric parameters correlating with secreted factors from SAT.

Anthropometric Parameter	Factor	R Value	*p* Value	*n*
Skeletal muscle	VEGF-A	−0.7667	0.02	9
IMF	TNF-α	0.6727	0.02	11

Abbreviations: IMF—intermuscular fat.

## Data Availability

The data presented in this study are available upon reasonable request from the corresponding author. The data are not publicly available as there are restrictions on data processing in line with participant consent.

## Supplementary Materials

The following are available online at https://www.mdpi.com/article/10.3390/metabo11110768/s1, Figure S1: Computed tomography assessment of body composition, Table S1: Changes in body composition from time of diagnosis to surgery, Table S2: Patient Characteristics.
