# Supplementary material for: Opposing Immune-Metabolic Signature in Visceral Versus Subcutaneous Adipose Tissue in Patients with Adenocarcinoma of the Oesophagus and the Oesophagogastric Junction
Source: Metabolites. 2021 Nov 10;11(11):768. doi: 10.3390/metabo11110768 (PMC8624269; doi:10.3390/metabo11110768)
Supplement: Supplementary file 1 [file metabolites-11-00768-s001.zip › Supplementary figure 1 081121.pdf]

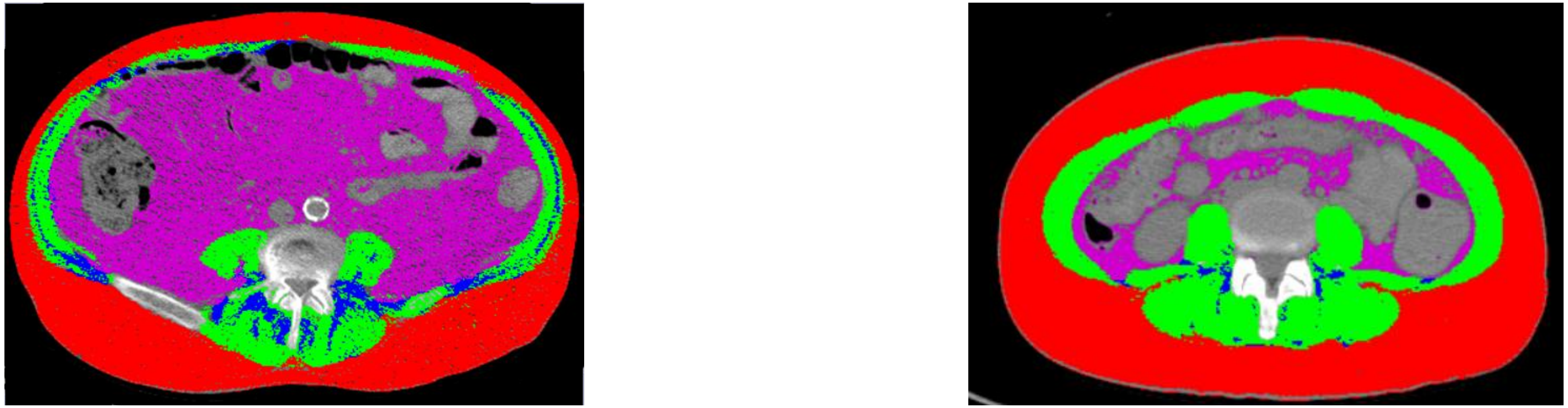

Supplementary figure S1. Computed tomography assessment of body composition
Abdominal CT images at L3 indicating subcutaneous adipose tissue in red, skeletal muscle in green, intermuscular fat in blue and visceral adipose tissue in pink. (**A**) Abdominal CT image at L3 from a viscerally obese sarcopenic patient. (**B**) Abdominal CT image at L3 from a non-viscerally obese non-sarcopenic patient.
