# Supplementary material for: Opposing Immune-Metabolic Signature in Visceral Versus Subcutaneous Adipose Tissue in Patients with Adenocarcinoma of the Oesophagus and the Oesophagogastric Junction
Source: Metabolites. 2021 Nov 10;11(11):768. doi: 10.3390/metabo11110768 (PMC8624269; doi:10.3390/metabo11110768)
Supplement: Supplementary file 1 [file metabolites-11-00768-s001.zip › Supplementary tables 081121.pdf]

Supplementary table S1. Changes in body composition from time of diagnosis to surgery

	**Diagnosis**	**At surgery**	***p* value**
**Total body fat mass (kg)**	23.03 ± 4.9	24.44 ± 7.75	>0.99
**Total body fat free mass (kg)**	52.27 ± 10.9	49.11 ± 10.54	0.0078
**Visceral fat area (cm^2)**	157.1 ± 123.5	148.7 ± 119.3	0.21
**Subcutaneous fat area (cm^2)**	163.5 ± 71.99	167.5 ± 100.7	>0.99
**Skeletal muscle (cm^2)**	154 ± 36.34	143.5 ± 35.15	0.0078
**Intermuscular fat (cm^2)**	11.69 ± 7.22	9.24 ± 3.67	0.68

Statistics performed on n=8 patients that received neoadjuvant treatment. Intermuscular fat (cm^2) only available for 7 patients.

Supplementary table S2. Patient Characteristics

n=12			Percent (%)
**Age**	Mean ± SD<br>Range	65.6 ± 11.8<br>46-83	
**Gender**	Male (n)<br>Female (n)	9<br>3	75<br>25
**Obesity status (pre-treatment)[a]**	Non-obese<br>Obese (n)	7<br>5	58.33<br>41.67
**Cancer**	Oesophageal adenocarcinoma (OAC) (n)<br>Oesophageal gastric junction (n)	6<br>6	50<br>50
**T stage**	T0 (n)<br>T1 (n)<br>T1a (n)<br>T1b (n)<br>T2 (n)<br>T3 (n)<br>T4b (n)	1<br>1<br>1<br>2<br>1<br>5<br>1	8.33<br>8.33<br>8.33<br>16.67<br>8.33<br>41.67<br>8.33
**N stage**	N0 (n)<br>N1 (n)<br>N2 (n)	6<br>5<br>1	50<br>41.67<br>8.33
**M stage**	Mx (n)	12	100
**Stage of differentiation[b]**	Moderate (n)<br>Moderate to poor (n)<br>Poor (n)	4<br>1<br>6	33.33<br>8.33<br>50
**Treatment received**	Surgery only (n)<br>FLOT + Surgery (n)<br>FOLFOX + Surgery (n)<br>CROSS + Surgery (n)	4<br>3<br>1<br>4	33.33<br>25<br>8.33<br>33.33
**TRG[c]**	1 (n)<br>2 (n)<br>3 (n)<br>4 (n)	1<br>1<br>4<br>2	12.5<br>12.5<br>50<br>25

[a] Obesity status determined at diagnosis by CT defined as visceral fat area >80.1 cm^2 for females and 163.8 cm^2 for males

[b] Stage of differentiation unavailable for one patient

[c] TRG available for 8 patients. Expressed as a % of patients with a TRG
